# Mitotic Proliferative Nodule Within a Giant Congenital Nevus: One Case Report and Updated Review

**DOI:** 10.3390/dermatopathology13030028

**Published:** 2026-06-23

**Authors:** Philippe Drabent, Nicolas Macagno, Sylvie Fraitag

**Affiliations:** 1 Department of Pathology, Hôpital Necker-Enfants Malades, APHP, 75015 Paris, France; 2 Department of Pathology, Centre Hospitalier Universitaire le Timone, APHM, 13385 Marseille, France

**Keywords:** children, congenital nevus, proliferative nodule, melanoma

## Abstract

Proliferative nodules (PNs) are benign, well-limited melanocytic proliferations that can occur within congenital nevi, particularly larger ones. Although they may mimic melanoma clinically and histologically, PNs are characterized by a monomorphic, well-defined cell population with peripheral blending with the adjacent nevus cells, and a lack of severe atypias, numerous mitoses (in most instances), necrosis, or inflammation. They generally present at birth or early childhood, and even with cytological atypia, they do not undergo malignant transformation. The risk of malignancy associated with a large/giant congenital nevus is low but increases with size and the presence of multiple satellite lesions. Diagnostic tools, including immunohistochemistry and, in selected cases, molecular techniques such as CGH-array or RNA-seq, can help differentiate atypical PNs from melanoma. Awareness of this entity and its diverse histological features is crucial to avoid over-diagnosis of malignancy and unnecessary interventions. Here we report a case of atypical PNs in a giant congenital nevus and discuss the literature.

## Simple Summary

Congenital nevi, particularly large or giant forms, may contain nodules that raise concern for melanoma. Distinguishing proliferative nodules from melanoma remains a major diagnostic challenge, as conventional histopathological features may partly overlap, particularly mitotic activity. We report a mitotically active proliferative nodule arising within a giant congenital nevus in a 7-week-old infant, showing an elevated mitotic rate and a high Ki67 index, yet with benign clinical evolution over 7 years. From this didactic case, we discuss the main useful features for the differential diagnosis between a proliferative nodule and melanoma in a congenital nevus. Notably, strong and diffuse 5-hmC expression supports the diagnosis of a benign proliferative nodule. CGH or NGS are mostly unnecessary and may be useful in selected worrisome cases. This review highlights the importance of integrating morphology, age-related context, and selected ancillary techniques in diagnosis and underscores the risk of overdiagnosis of melanoma, with significant implications for clinical management.

## 1. Case Report

A 7-week-old infant was born with a giant abdominal congenital nevus measuring 14 × 9 cm, with about 10 satellite lesions. He underwent surgery for a nodule, present at birth, within the giant congenital nevus. The nodule was firm, round, of the same pigmentation as the surrounding nevus clinically, with no ulceration, and measured 1 cm in diameter. Gross examination of the nodule revealed a homogeneous cut section and a fairly lighter pigmentation compared to the adjacent nevus.

**Figure 1:**
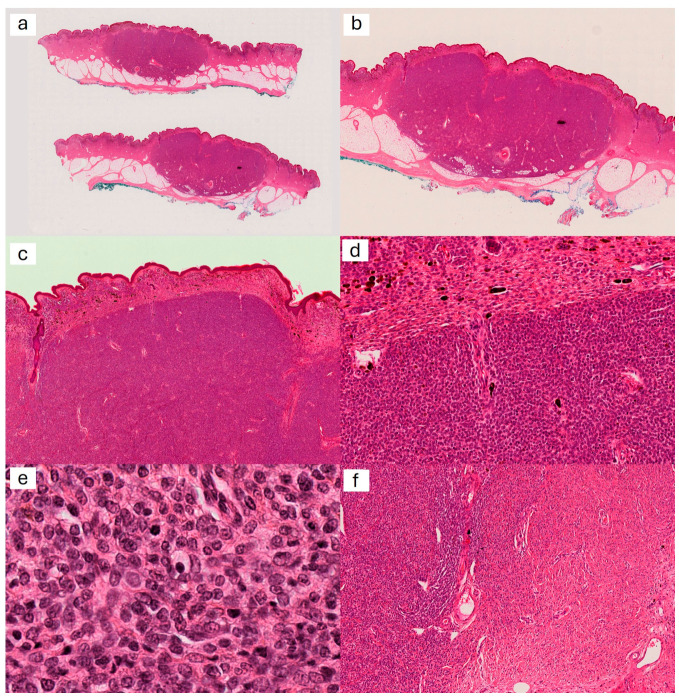
Mitotic proliferation nodule in a giant congenital naevus. Histopathology: (**a**) dermal and subcutaneous nodular cellular lesion; (**b**,**c**) lesion appears relatively well-circumscribed compared to the adjacent naevus, with no areas of necrosis or inflammation; (**d**) densely cellular nodule; (**e**) the cells are epithelioid and show mild anisokaryosis; (**f**) in reality, the nodule is not sharply circumscribed, with areas where the nodule cells blend with those of the adjacent naevus. This figure is from the pathology department at Necker-Enfants Malades Hospital and illustrates the corresponding pathology.

Microscopic examination showed a relatively well-defined dermo-hypodermic nodular lesion (Figure 1a–c) situated within a congenital-looking nevus. There were no areas of necrosis or inflammation. The nodule contained melanocytic cells with mild anisokaryosis, nuclei being only slightly larger than those of the adjacent nevus (Figure 1e). The cellular density (Figure 1d) was markedly increased compared to the nevus. Mitotic figures were present with up to 7 mitoses per mm^2^, including deep-seated mitoses. At the periphery of the nodule, cells were clearly merging with those of the adjacent nevus (Figure 1f).

**Figure 2:**
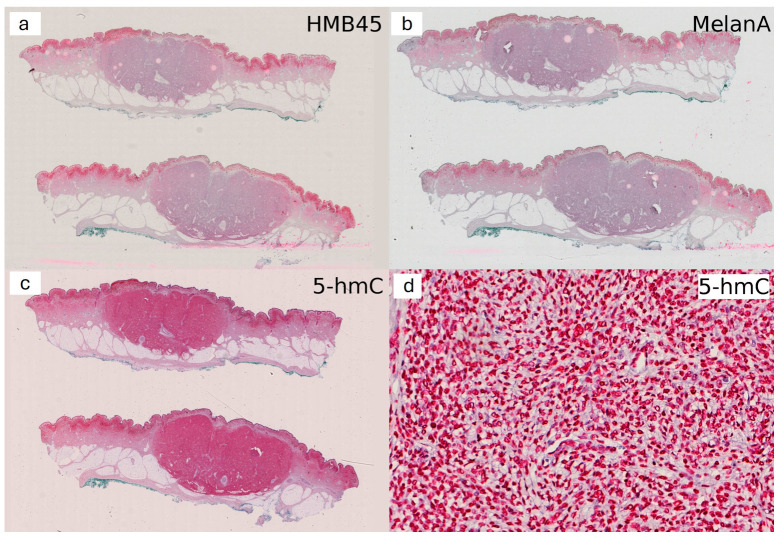
Immunohistochemistry: (**a**) HMB45; (**b**) MelanA. HMB45, and anti-MelanA antibodies weakly but uniformly stain the nodule. The adjacent naevus is much more strongly stained; (**c**,**d**) 5-hmC. The anti-5-hmC antibody strongly and uniformly stains all the nuclei of the nodule cells. This figure is from the pathology department at Necker-Enfants Malades Hospital and illustrates the corresponding pathology.

**Figure 3:**
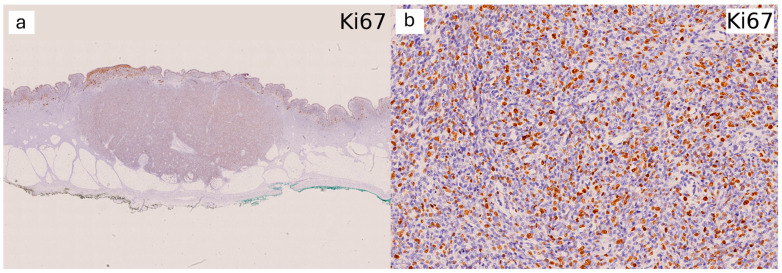
Immunohistochemistry: Ki67. It shows a very high proportion of stained nuclei (cells in the cycle), reaching 60% in certain areas (**a**,**b**). This figure is from the pathology department at Necker-Enfants Malades Hospital and illustrates the corresponding pathology.

HMB45 (Figure 2a) and MelanA (Figure 2b) were very faintly positive in the nodule, whereas the adjacent nevus was much more strongly marked. P16 was negative. 5-hmC stained all the nuclei of the cells in the nodule in a strong, homogeneous manner (Figure 2c,d). Ki67 showed a very high proportion of cells in the cycle, reaching 60% in hot-spot areas (Figure 3).

The diagnosis of a mitotic proliferative nodule within a giant congenital nevus was made. A 7-year follow-up did not show any recurrence.

## 2. Discussion

### 2.1. Congenital Nevus

**Figure 4:**
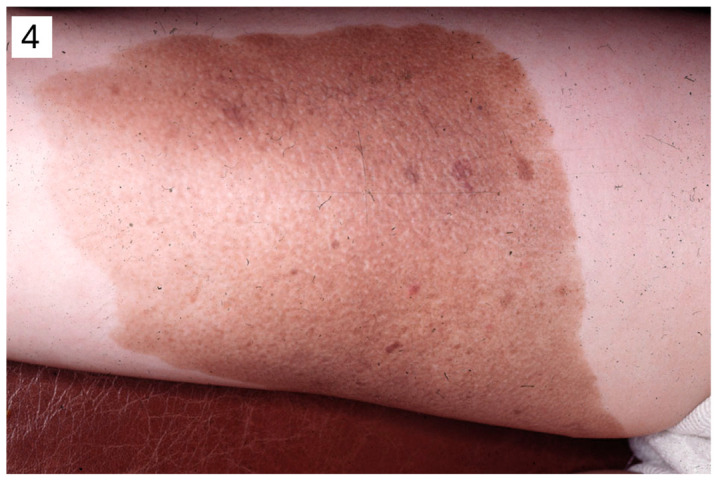
Congenital naevus. Clinical aspects (Hôpital Necker-Enfants Malades). Intermediate-sized congenital naevus. This figure is from the dermatology department at Necker-Enfants Malades Hospital and illustrates the corresponding pathology.

Congenital nevi (CN) are benign melanocytic proliferations originating from the neural crest, present at birth or, less frequently, occurring within the first few weeks of life. CN are classified into four groups based on their expected adult size (“projected adult size”):

– Small congenital nevus: diameter less than 1.5 cm in adulthood;
– Intermediate-sized congenital nevus (Figure 4): diameter between 1.5 and 19.9 cm in adulthood;
– Large congenital nevus: diameter between 20 and 40 cm in adulthood;
– Giant congenital nevus: diameter greater than 40 cm in adulthood.

CN do not grow uniformly across different areas of the body. For instance, a CN measuring 10 cm at birth will measure as follows in adulthood:

– If located on the head, 20cm;
– If located on the trunk or upper limb, 25 to 27 cm;
– If located on the lower limb, 30 cm.

CN are very common, affecting 1 to 2% of newborns. While most of them are small, 1 in 1000 individuals has an intermediate-sized nevus, 1 in 20,000 has a large nevus, and only 1 in 500,000 has a giant nevus [1]. They result from early post-zygotic mutations of melanoblasts [2]. These are “mosaic RASopathies”. The same mutation is found in the skin and in the central nervous system if it is affected [3]. Genetic features of CN are discussed in more detail later.

CN can occur anywhere on the body, but they are more frequently located on the trunk and lower limbs than on the head and neck or upper limbs. They are round, oval, or quadrangular in shape, and can be macular or papular, with either smooth or verrucous surfaces. Some may resemble “spilus nevi”.

**Figure 5:**
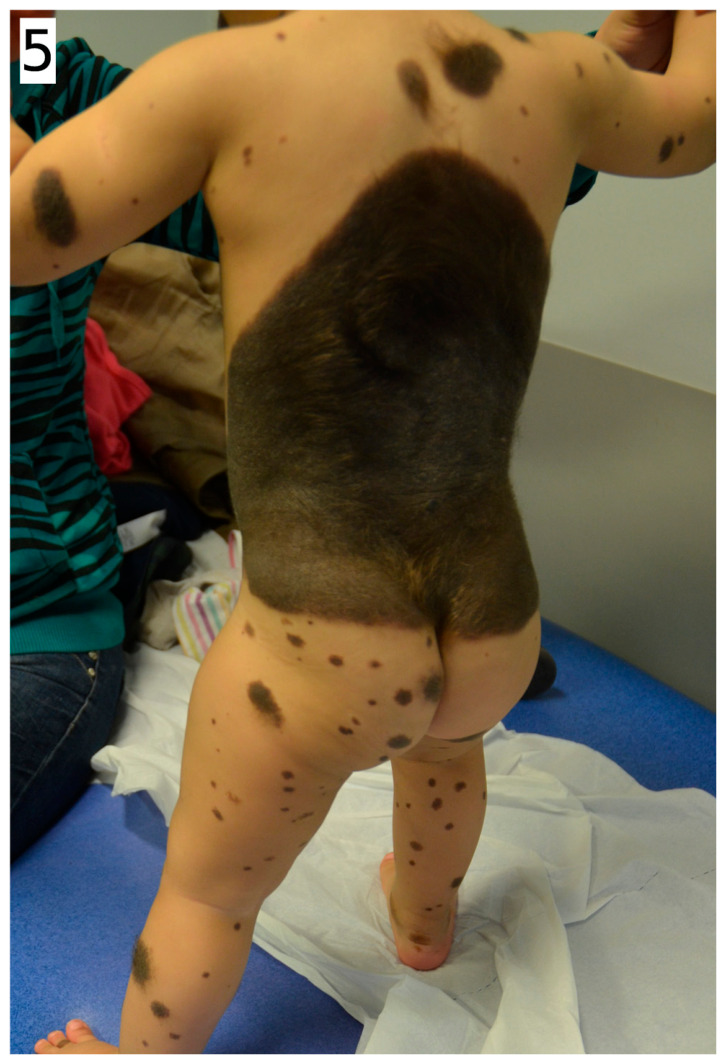
Congenital naevus. Clinical aspects (Hôpital Necker-Enfants Malades). Giant congenital naevus of the trunk with numerous satellite naevi. This figure is from the dermatology department at Necker-Enfants Malades Hospital and illustrates the corresponding pathology.

**Figure 6:**
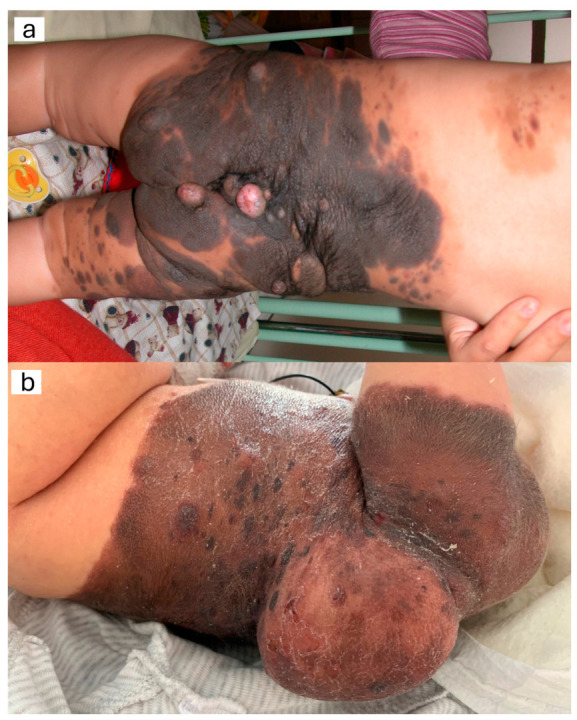
Congenital naevus. Clinical aspects (Hôpital Necker-Enfants Malades). Giant congenital naevi: (**a**) multinodular lesion; (**b**) giant congenital naevus with a huge tumour. This figure is from the dermatology department at Necker-Enfants Malades Hospital and illustrates the corresponding pathology.

**Figure 7:**
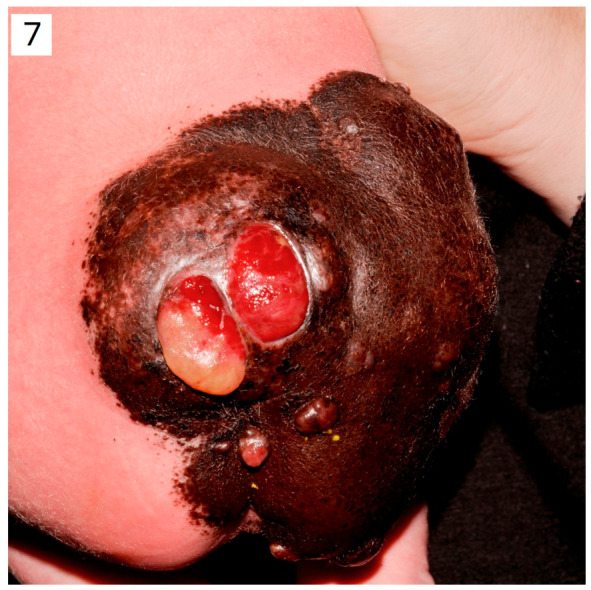
Large congenital naevus (Hôpital Necker-Enfants Malades). Clinical aspects. Thick lesion on the trunk with several ulcerated nodules. This figure is from the dermatology department at Necker-Enfants Malades Hospital and illustrates the corresponding pathology.

Large/giant CN are often very polymorphic and heterochromatic, with verrucous surfaces. They are frequently accompanied by small distant nevi known as satellites (Figure 5). They may contain nodules or even tumours (Figure 6). Over time, these nevi may thicken, lighten, or become covered with hair. New nodules may appear, and existing nodules may ulcerate (Figure 7). When there is involvement of the leptomeninges by mutated melanocytic cells, this is referred to as neurocutaneous melanocytosis or “congenital nevus syndrome”. This involvement can be seen on an MRI if the examination is performed early. It can lead to delays in psychomotor development or seizures, though it may be asymptomatic [4].

The main risk is the development of cutaneous or leptomeningeal melanoma [3,4,5]. The risk of malignancy, taking into account all sizes of CN, is between 0.7% and 1.25%. In the case of a large or giant CN, the risk of developing a melanoma is between 3% and 8%, proportional to the size of the nevus, and higher if the congenital nevus is located on the trunk and is accompanied by “multiple satellite nevi.” However, this risk is also higher if there are at least 2 congenital nevi, regardless of their size, or if the meningoencephalitic MRI is abnormal [5].

### 2.2. Proliferative Nodules in a CN

**Figure 8:**
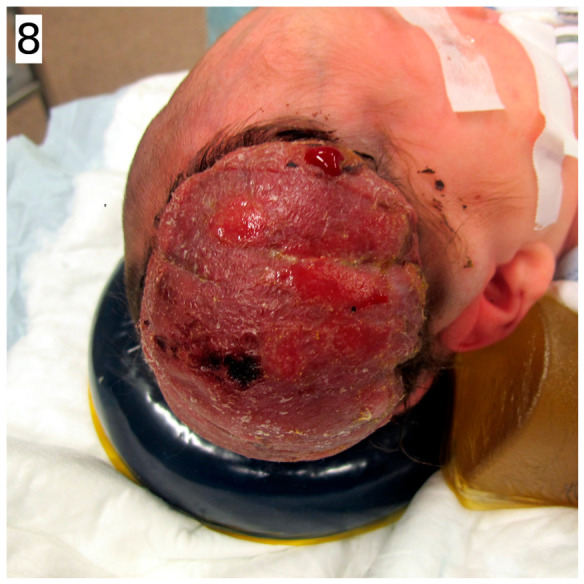
Large congenital naevus (Hôpital Necker-Enfants Malades). Clinical aspects. Scalp lesion with a large proliferation nodule. This figure is from the dermatology department at Necker-Enfants Malades Hospital and illustrates the corresponding pathology.

Proliferative nodules (PNs) are well-limited, compact clusters of benign melanocytic cells within a conventional CN. Clinically, they can present as papules, nodules, plaques, or tumours, and are more commonly seen in large CN, but they can also appear in small CN [6]. PNs are generally present at birth but may appear later, even in adulthood, which can lead to a challenging differential diagnosis with melanoma [7]. They may be isolated or multiple, more or less pigmented, and of a different colour from the adjacent CN, sometimes ulcerated. Their size varies, ranging from a few millimetres to several centimetres (Figure 8). Finally, they may sometimes regress spontaneously but are generally stable, non-progressive, and never transform into melanoma, even when they are histologically atypical. However, PNs can mimic melanoma.

**Figure 9:**
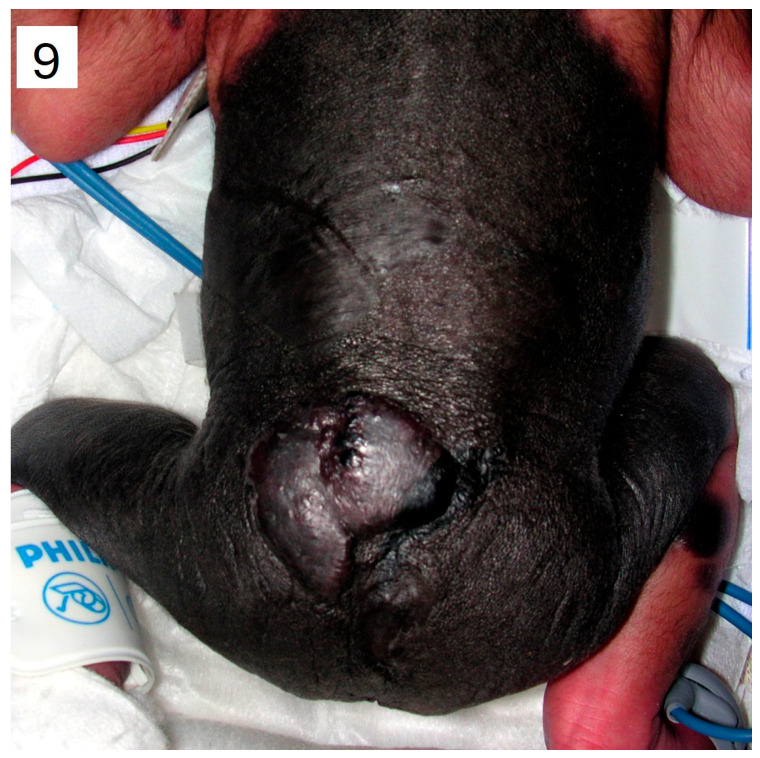
Large congenital naevus (Hôpital Necker-Enfants Malades). Clinical aspects. Lumbar lesion with a neurocristic hamartoma. This figure is from the dermatology department at Necker-Enfants Malades Hospital and illustrates the corresponding pathology.

In addition, a number of hamartomatous or other tumour-like lesions can also develop within large and giant CN, such as sarcomas (rhabdomyosarcomas, among others) [8], neurocristic hamartomas (Figure 9), which are foci of mesenchymal, muscular, adipose, chondroid, or osseous differentiation [9], and foci of marked neuroid differentiation (such as neurofibroma, sometimes with tactile corpuscles, or neuroblastoma, or hybrid neural tumour such as schwannoma/perineurioma) [10]. All these lesions can present as nodules or tumours.

**Figure 10:**
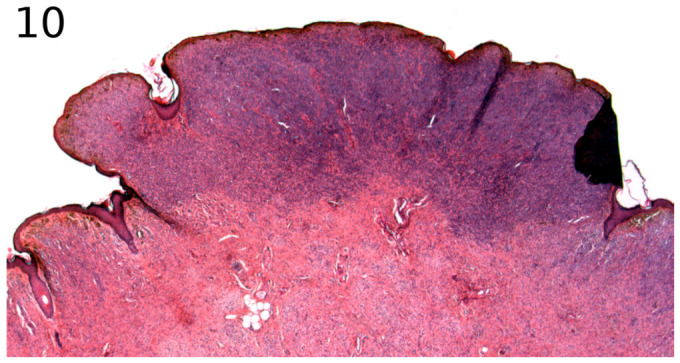
Histopathology (H&E). Superficial hypercellularity focus. Lesion located in the superficial dermis. This figure is from the pathology department at Necker-Enfants Malades Hospital and illustrates the corresponding pathology.

**Figure 11:**
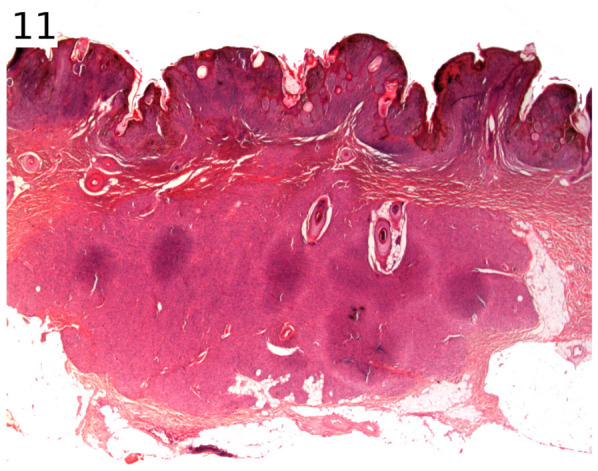
Histopathology (H&E). Proliferation nodule. Lesion located in the deep dermis and hypodermis. This figure is from the pathology department at Necker-Enfants Malades Hospital and illustrates the corresponding pathology.

**Figure 12:**
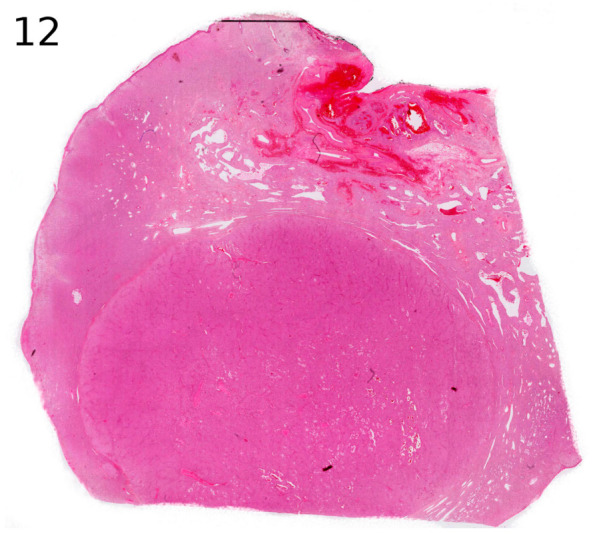
Histopathology (H&E). Proliferation nodule. Lesion clearly distinct from the adjacent congenital naevus due to its compact nature. This figure is from the pathology department at Necker-Enfants Malades Hospital and illustrates the corresponding pathology.

**Figure 13:**
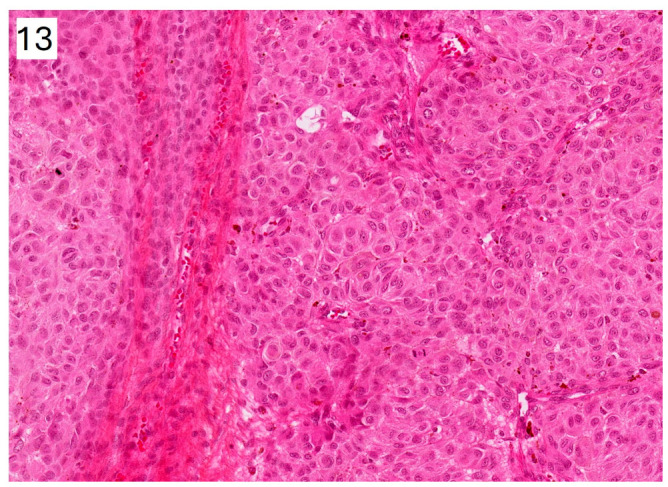
Histopathology (H&E). Proliferation nodule. The nodule cells are epithelioid and here are achromatic. This figure is from the pathology department at Necker-Enfants Malades Hospital and illustrates the corresponding pathology.

**Figure 14:**
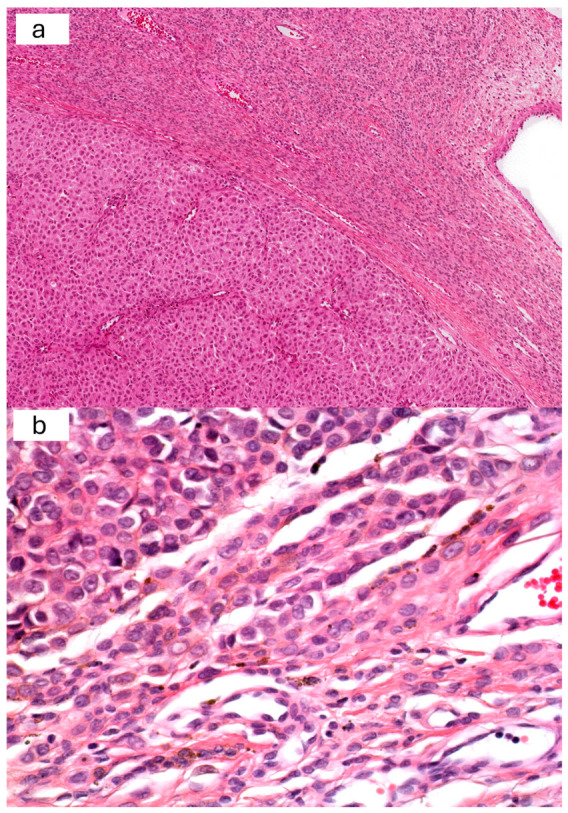
Proliferation nodule. Histopathology (H&E): (**a**) the nodule appears well-defined but, in reality, shows blending with the adjacent naevus; (**b**) this “mixing” of populations is more evident at high magnification. This figure is from the pathology department at Necker-Enfants Malades Hospital and illustrates the corresponding pathology.

**Figure 15:**
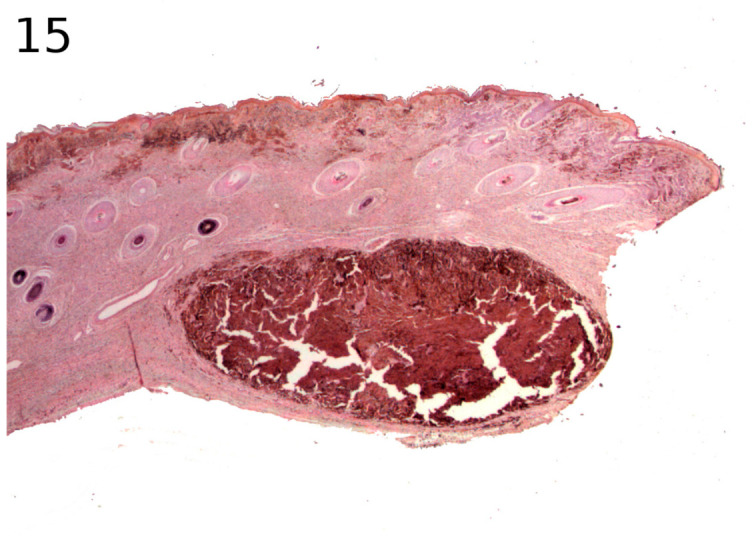
Proliferation nodules. Histopathology (H&E). Pigmented proliferation nodule. The lesion contrasts with the adjacent congenital naevus, which is very lightly pigmented. This figure is from the pathology department at Necker-Enfants Malades Hospital and illustrates the corresponding pathology.

**Figure 16:**
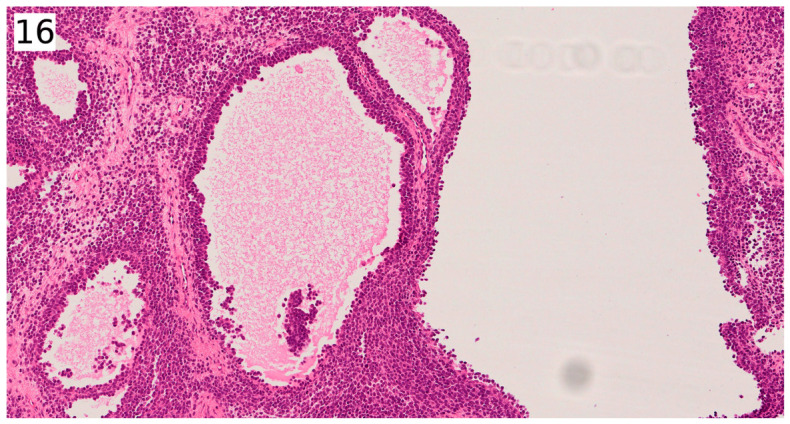
Proliferation nodules. Histopathology (H&E). Adenoid proliferation nodule. This figure is from the pathology department at Necker-Enfants Malades Hospital and illustrates the corresponding pathology.

From a histological point of view, PNs can be superficial, located just below the epidermis, and are then referred to as “superficial hypercellularity foci” (Figure 10). When they are located deeper, in the reticular dermis and/or subcutis, they are rather called “proliferative nodules” (Figure 11). They appear as compact foci of cells that contrast with the adjacent nevus cells. The compact and well-defined nature of these foci, visible at low magnification, makes them easily identifiable at first glance (Figure 12). Their histological appearance is variable. In the vast majority of cases, the cells of the nodule are regular, only slightly larger than those of the adjacent nevus and monomorphic (Figure 13). The nodule appears well-defined but, under high magnification, its cells are seen to blend into those of the adjacent nevus (Figure 14a,b). There are generally fewer than 2 mitoses per mm^2^, and the proliferation index is generally low. The cells are most often epithelioid, hyperpigmented, or, conversely, achromic (Figure 15). One may also observe nevus-like, spindle-shaped, or ballooned cells, and adenoid architecture (Figure 16), or even storiform patterns.

Different cytological variants of proliferative nodules have been described:

– Small, rounded, blue melanocytes (“melanoblasts”): In newborns or very young children, this variant is very likely to be misdiagnosed as melanoma. It has no particular pattern, and the cells look like lymphocytes, but they may be atypical and mitotically active. The absence of necrosis is helpful, as well as the peripheral blending of cells, but it may be indistinguishable from a melanoma, and molecular techniques or at least a CGH-array are recommended.
– A Spitz nevus-like appearance: It is usually a hypopigmented nodule with large epithelioid melanocytes harbouring a vesicular nucleus and prominent nucleolus, similar to typical Spitz morphology. In typical PNs, cells are monomorphous and mitoses are low, which easily rules out melanoma. However, in atypical PNs, cells have large hyperchromatic nuclei with dense chromatin, some mitoses, and even necrotic cells in some instances. A good clue is the fact that, although atypical, the cells are all atypical in the same way, meaning there is some kind of homogeneity. Even if there are mitoses, they are not numerous. If not excised, this kind of nodule evolves, with time, to a more classical PN.
– A blue nevus-like appearance: This is a hyperpigmented nodule with pigmented bipolar dendritic cells on histology and melanophages. The lack of nuclear atypia, absence of necrosis, and low mitotic rate are in favour of a benign PN. A differential diagnosis that should not be missed is that of a melanophage nodule, composed exclusively of melanophages from a completely regressed melanoma. Although rare, this occurrence should be known. A macrophage marker (CD68) with red staining (alkaline phosphatase) is useful.
– A “deep penetrating nevus” (DPN) appearance (now called WNT-activated melanocytoma = WAM): This kind of nodule is very similar to a typical WAM, with large spindle or oval melanocytes with some pleomorphism, mixed with melanophages in a “grid” or “checkerboard” pattern. There may be large nucleoli. The differential diagnosis with melanoma is based upon the benign architecture of the nodule (symmetry, sharp circumscription, evenly spaced melanophages), the absence of necrosis, and low mitotic index.
– A rhabdoid (or plasmacytoid) appearance: This cytology can be very worrying. In favour of a benign nodule are the cellular monomorphism, maturation of cells at the periphery of the nodule, and architecture in small nests or clusters in the nodule.
– A neural appearance: Characterized by a schwannian differentiation, which may be interpreted as a neurofibroma. This cytology is not worrisome. Of note, compound CN-neurofibromas have been described.
– Other “divergent” differentiations (muscular, adipocytic, chondroid, osteoid).

All those variants raise the question of the molecular genetics behind these PNs. It is probable that WAM-like (DPN-like) PNs arise from a second molecular anomaly involving *CTNNB1* or *APC*, just as has been proven for WAM in a common nevus setting. Similarly, it would be very interesting to know if typical “blue-type” alterations can be found in blue nevus-like PNs, typical Spitz alterations in Spitz nevus-like PNs, or even *PRKAR1A* or *BAP1* inactivation in a subset of PNs, as described in common nevi. For the time being, there is no molecular proof of this concept.

**Figure 17:**
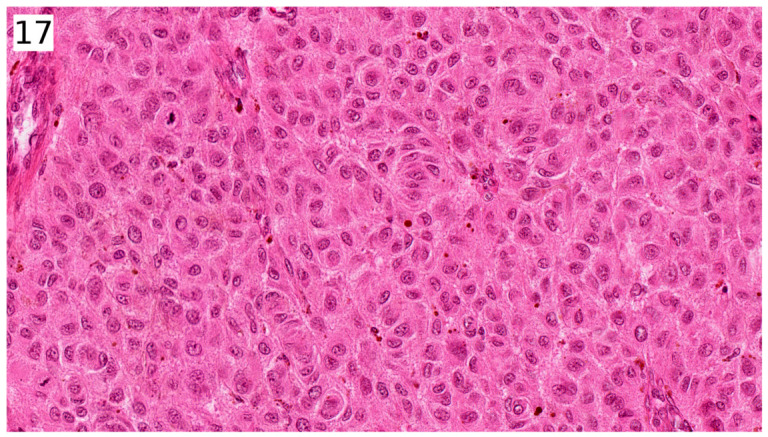
Proliferation nodules. Histopathology (H&E). Proliferation nodule. The cells may have pleomorphic nuclei and show a few mitotic figures, particularly if the patient is an infant. This figure is from the pathology department at Necker-Enfants Malades Hospital and illustrates the corresponding pathology.

Rarely, the nodule can be atypical with a clear boundary from the adjacent nevus (without a “transition” image), cytological and nuclear atypia, or mitoses. The cells may be atypical and large, with pleomorphic nuclei (Figure 17). A good clue may be the presence of different cytological areas or clones within the nodule, reminiscent of the typical heterogeneous pleomorphism of some melanomas. The epidermis may be ulcerated. The proliferation index may be elevated, as in the present case; however, such mitotic activity is usual in neonates and young infants, and the younger the child, the more mitotic the nodule. In fact, just like adjacent congenital nevi, PNs may be more atypical and mitotic when examined early in life. Thus, the child’s age should be taken into account when examining these lesions microscopically. After the neonatal period, it is rare for the proliferation rate to exceed 15%. All these cases are referred to as “atypical proliferative nodules” [11]. Importantly, even when atypical, PNs are always benign and do not transform.

These atypical PNs may pose a differential diagnostic challenge with melanoma developing within a large/giant CN (see Table 1), particularly since this type of melanoma is also nodular in morphology and develops deeply, away from the epidermis. In favour of PNs are the absence of necrosis, inflammation, or destruction of adnexal structures.

**Figure 18:**
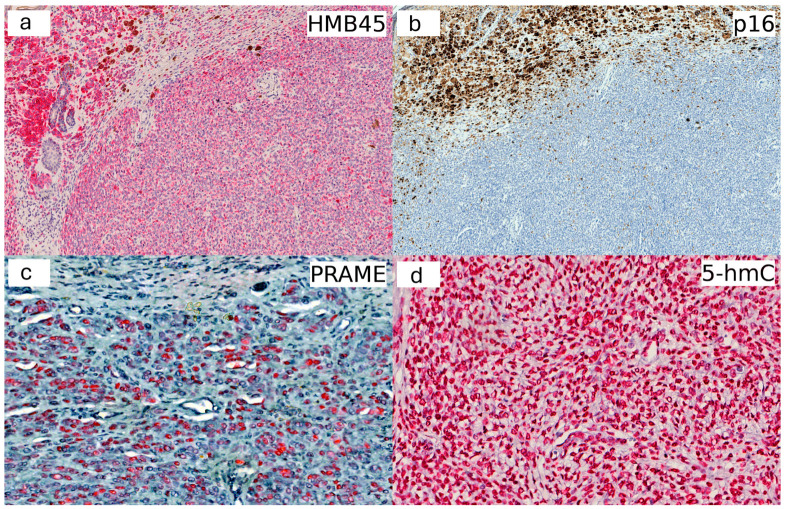
Typical proliferation nodule. Immunohistochemistry: (**a**) HMB45. The nodule is faintly positive; (**b**) p16. The nodule is negative, which is common; (**c**) PRAME. Here, 60% of the nodule nuclei are stained. Therefore, care must be taken in interpreting this marker; (**d**) 5-hmC. The nodule is strongly positive. This figure is from the pathology department at Necker-Enfants Malades Hospital and illustrates the corresponding pathology.

Usual immunohistochemical markers such as HMB45 (Figure 18a), MelanA, or p16 (Figure 18b) are of no help in the differential diagnosis. Most PNs display intense homogeneous staining for HMB45, but melanomas are also positive for HMB45, with staining more often homogeneous in childhood melanomas than in adult-onset melanomas. Most PNs, as well as all childhood-onset melanomas, can also be strongly positive for p16 [12].

The PRAME marker (PReferentially expressed Antigen in MElanoma) seems useful, but it should be interpreted with caution, as although it is always positive in melanomas, it can also be positive in PNs (Figure 18c). Conversely, a negative PRAME result supports a PN diagnosis [13,14].

Two markers are of particular interest: the methylation markers H3K27me3 and 5-hmC (Figure 18d). Their nuclear expression is homogeneous and strong in proliferative nodules, while it is reduced or absent in melanoma [15,16,17].

FISH analysis does not help in this differential diagnosis. Indeed, numerical chromosomal abnormalities and instabilities [12] can be found in both PNs and melanomas developing from congenital nevi in childhood. CGH-array seems more useful if it reveals chromosomal breakpoints or gains, which are signatures of melanoma [18,19,20]. Pangenomic RNA-seq is simpler to implement and faster. Mass spectrometry could be a promising method, but it is not available everywhere [21].

### 2.3. Genetic Considerations

CN may have different genetic signatures leading to the activation of the MAP kinase signalling pathway. Small or intermediate-sized unique CN are most often *BRAF*-mutated, sometimes for *NRAS* or *GNAQ*. Large and giant CN and multiple CN (≥2 congenital nevi), in 80% of cases, are associated with mosaicism in codon 61 of the *NRAS* gene [22,23,24]. Chromosomal rearrangements involving other MAP kinase pathway genes, such as *RAF1*, have also been reported [7,25,26,27].

### 2.4. Practical Guidelines for Pathologists (See Table 2)

In the case of a superficial or deep non-atypical nodule, immunohistochemical markers are unnecessary, and simple excision is recommended.

**Table 2:** Management of a proliferative nodule in a congenital naevus.

Superficial or Deep, Non-Atypical Nodule	Atypical Nodule (Cytonuclear Atypia, Mitoses)
IHC: unhelpful	- IHC: Ki67, 5-hmC or H3K27me3, PRAME (interpret with caution) - CGH array/RNA-seq - Mass spectrometry
Simple excision, nothing more!	Large excision, regular clinical follow-up

In the case of an atypical PN (with cytological-nuclear atypia and mitoses), malignancy must be ruled out.

Targeted immunohistochemistry (Ki67, 5-hmC or H3K27me3, or even PRAME) and more sophisticated techniques in some cases, such as comparative genomic hybridization (CGH-array) and pangenomic RNA-seq, will be used. The opinion of an expert may be helpful.

In cases of atypical nodules, some diagnostic uncertainty may persist, making clinical follow-up of paramount importance. The absence of local recurrence or regional metastasis provides definitive evidence of benignity.

## 3. Conclusions

It is important to be aware of the existence of proliferative nodules in congenital nevi and not confuse them with melanoma, which is much rarer. Most often, a simple histological examination is sufficient to diagnose a proliferative nodule, a lesion that is always benign.

## Figures and Tables

**Table 1:** Criteria for differentiating a proliferation nodule from a melanoma developed within a congenital naevus during childhood.

Criteria	Proliferation Nodule Within a Congenital Nevus	Melanoma Within a Congenital Nevus
**Morphology**	Peripheral cellular blending between nevus and nodule	Well-limited nodule, no blending (clone)
	No or discrete cytonuclear atypia	Marked cytonuclear atypia
	No or rare mitoses (<2/mm^2^) (not applicable in neonates)	Numerous mitoses, often atypical
	No necrosis nor inflammation	Necrosis, Inflammation
**IHC**	Ki67 (MIB1) < 15%	Ki67 (MIB1) > 40%
	HMB45 + homogeneous, p16 + (sometimes negative)	HMB45 + homogeneous, p16 + (sometimes negative)
	PRAME negative (sometimes positive)	PRAME positive
	H3K27me3 or 5-hmC positive	H3K27me3 or 5-hmC negative or decreased
**FISH**	Numerical abnormalities	Numerical abnormalities
**CGH array**	Numerical chromosomal abnormalities	Gains or losses of fragments of chromosomes

## Data Availability

No new data were created or analyzed in this study. Data sharing is not applicable to this article.
